# Effect of rapid maxillary expansion on nasal cavity assessed with cone-beam computed tomography

**DOI:** 10.1590/2177-6709.25.3.039-045.oar

**Published:** 2020

**Authors:** Luciana Duarte Caldas, Wilton M. Takeshita, André Wilson Machado, Marcos Alan Vieira Bittencourt

**Affiliations:** 1 Universidade Federal da Bahia, Faculdade de Odontologia (Salvador/BA, Brazil).; 2 Universidade Federal do Sergipe, Faculdade de Odontologia, Departamento de Radiologia Oral (Aracaju/SE, Brazil).; 3 Universidade Federal da Bahia, Faculdade de Odontologia, Departamento de Ortodontia (Salvador/BA, Brazil).

**Keywords:** Maxillary expansion, Nasal cavity, Cone-beam computed tomography

## Abstract

**Introduction:**

Rapid maxillary expansion (RME) is assumed as a well established procedure; although, some effects on facial complex are not yet fully understood.

**Objective:**

The aim of this research was to verify, using cone-beam computed tomography, the effect on linear dimensions of the nasal cavity.

**Methods:**

Sample consisted of twenty patients aged 7 to 16 years, with skeletal deformity that justified the use of CT scans, and who required the RME as part of the orthodontic treatment planning. Scans were taken before clinical procedures were performed (T_0_) and after stabilizing the expander screw (T_1_). Dolphin Imaging v. 11.5 3D software was used to measure six areas on nasal cavity: three at the anterior portion (upper, middle, and lower) and other three at the posterior portion (also upper, middle, and lower). Data were statistically treated using Shapiro-Wilk test to verify normality. Differences between T_0_ and T_1_ were calculated using the Spearman correlation and paired Student’s *t*-test, with a significance level of 5%.

**Results:**

All linear measurements presented a significant increase (*p*< 0.05) after RME, both in the anterior and posterior regions, suggesting some parallelism on the opening pattern, especially at the lower portion (*p*< 0.001).

**Conclusions:**

RME was able to significantly modify the internal dimensions of the nasal cavity.

## RESUMO

**Introdução:**

A expansão rápida da maxila (ERM) é um procedimento bem estabelecido. Entretanto, alguns efeitos no complexo facial ainda não foram completamente compreendidos.

**Objetivo:**

O objetivo do presente estudo foi verificar o efeito da ERM nas medidas lineares da cavidade nasal, utilizando a tomografia computadorizada de feixe cônico (TCFC).

**Métodos:**

A amostra foi composta por 20 pacientes da Universidade Federal da Bahia, com idades entre 7 e 16 anos, com deformidades esqueléticas que justificavam o uso da TCFC e que necessitavam da ERM como parte do tratamento ortodôntico. As imagens tomográficas foram realizadas antes dos procedimentos clínicos (T_0_) e após estabilização do parafuso expansor (T_1_). O *software* Dolphin Imaging v. 11.5 3D (Dolphin, Chatsworth, CA, EUA) foi utilizado para mensurar seis áreas na cavidade nasal, três delas na região anterior (superior, média e inferior) e outras três na região posterior (também superior, média e inferior). Os dados foram trabalhados estatisticamente, utilizando o teste de Shapiro-Wilk para avaliar a normalidade. Diferenças entre T_0_ e T_1_ foram calculadas usando a Correlação de Spearman e o teste t de Student pareado, usando um nível de significância de 5%.

**Resultados:**

Todas as mensurações lineares apresentaram um aumento significativo (*p*< 0,05) após a ERM, tanto na região anterior quanto na posterior, sugerindo algum paralelismo no padrão de abertura, principalmente na porção inferior (*p*< 0,001).

**Conclusão:**

A ERM foi capaz de modificar significativamente as dimensões internas da cavidade nasal.

## INTRODUCTION

Rapid maxillary expansion (RME) is an orthodontic and orthopedic procedure that has been used for over 100 years for correction of maxillary atresia.^1^ ^-^ ^4^ RME orthopedic action basically occurs by opening the median palatine suture, which guarantees a real skeletal gain in transverse dimension of the maxilla. Additionally, it also has the potential to alter the internal dimensions of the nasal cavity, promoting reduction of nasal resistance, increase in air flow and even a favorable change at the patient breathing pattern.^1^ ^,^ ^5^ ^-^ ^13^

Most studies related to this therapy have been conducted on the basis of occlusal and cephalometric radiographs, especially due to the fact that these exams are important for orthodontic treatment planning and are commonly part of the set of exams requested for this purpose.^1^ ^,^ ^5^ ^,^ ^8^ ^,^ ^14^ ^-^ ^20^ Nevertheless, in spite of being capable of providing great information, radiographs are two-dimensional images of a three-dimensional structure.^21^

Although many studies have been conducted about RME, there is still no consensus in the literature about the real effects of this procedure on the respiratory pattern.^6^ ^,^ ^9^ ^,^ ^11^ ^,^ ^13^ ^,^ ^17^ ^,^ ^22^ ^-^ ^25^ Methodological difficulties and lack of standardization of research evaluating the morphological changes that actually occur in the nasal cavity are common limitations. Currently, with the advent of cone beam computed tomography (CBCT), these problems have been minimized, since the CBCT enables acquisition of efficient and precise reproductions of anatomical structures.^26^ ^-^ ^28^ CBCT has been introduced on dental literature as an innovation in the way of acquiring volumetric images,^12^ with subsequent multiplanar reconstruction.^24^

A great number of researches evaluating the effects of RME using CBCT have been developed over the last few years,^28^ ^-^ ^33^ most of them focusing on volumetric changes,^10^ ^,^ ^12^ ^,^ ^23^ ^,^ ^25^ ^-^ ^27^ ^,^ ^34^ ^-^ ^38^ although it is also very important to analyze changes in linear dimensions. In this context, methods that allow a more objective analysis of linear changes induced by expansion are anticipated. Therefore, the aim of this research was to verify the effect of RME on linear dimensions of the nasal cavity using CBCT.

## MATERIAL AND METHODS

The present study was performed in accordance with the Code of Ethics of the World Medical Association (Declaration of Helsinki) and the Ethics Board of the Brazilian Ministry of Health (Resolution CNS/MS 466/2012) for research involving humans. This project was approved by the independent Ethics Committee of the School of Dentistry of Federal University of Bahia (Protocol #26/12) and registered with SISNEP FR 459942, CAAE 0028.0.368.000-11. The privacy rights of all subjects were observed, no identifying information of them was used, and written informed consent was obtained from all participating parents or legal guardians.

For this research, 20 patients ranging from 7 to 16 years of age were selected among those who sought the Federal University of Bahia orthodontics postgraduation program. The inclusion criteria involved: normal general health condition, no previous orthodontic treatment, no active caries and/or periodontal disease, need for RME as part of orthodontic treatment planning, and a vertical (hypo or hyperdivergent) or sagittal (Class II or Class III skeletal pattern) dentofacial deformity that would justify requesting a CBCT. Based on the literature^36^ and on the pilot study performed, the sample size was calculated (Table 1).

**Table 1:** Sample calculation based on literature^36^ and pilot test.

Sample calculation	
Standard deviation of difference	1.21
Average of the differences (after and before)	1.41
Level of significance	5%
Power of test	95%
Required sample size for each group	19

Selected patients were submitted to a new clinical exam, and before insertion of any accessory, a CBCT of the whole skull was taken (T_0_). RME was performed with a Haas expansion appliance, with bands on the permanent first molars, and depending on the patient’s stage of dental development, on the first premolars or primary first molars. 

Treatment included an active stage, which released lateral forces, and a passive stage of splinting. The active stage started 24 hours after appliance setting, and involved activating the screw twice a day. The activation stage lasted from two to four weeks, depending on the amount of expansion desired. At the end of this stage, the screw was stabilized with 0.012-in ligature wire (Morelli, Sorocaba/SP, Brazil), and a new tomography was taken (T_1_), with the aim of verifying the impact of expansion in all skeletal disharmonies presented by the patients. Appliances remained without activation for six months, while reorganization of maxillary suture was processed.

In order to obtain the CBCT, an i-CAT^®^ (Imaging Sciences International, Hatfield, PA, USA) equipment was used, with acquisition protocol configured at 120 kVp, 30 mA, voxel of 0.2 mm, FOV of 20*x*25 cm, acquisition time of 40 seconds, with an effective radiation dose of approximately 69 µSv. Patients were placed with Frankfort plane oriented parallel to the ground and with maximum habitual intercuspidation. In all patients, special attention was addressed to the position of the tongue, which had to be positioned on the central incisal papilla throughout the entire exam. DICOM (Digital Imaging and Communications in Medicine) files were imported and three-dimensional (3D) reconstructions of maxillary structures were performed using the Dolphin Imaging software v. 11.5 Premium (Dolphin, Chatsworth, CA, USA). After 3D reconstructions, the nasal cavity was delimited and the linear dimensions were calculated. 

Before taking measurements, it was necessary to standardize the position of the head, according to axial, coronal and sagittal planes, selected in both phases of the study. In lateral views, the right Orbital and Porion points were located and positioned in order to leave Frankfort plane coinciding with the software horizontal line. In frontal views, equivalent points in right and left zygomatico-frontal sutures were also marked and positioned in such a way to coincide with the software horizontal line, and the software median line positioned exactly in the patient’s median line.

**Figure 1:**
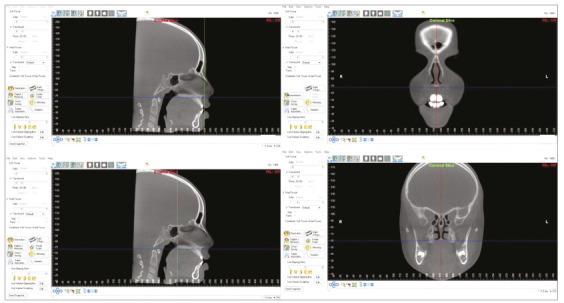
Standardization of positioning of the digital image of the head, in sagittal and coronal sections, using the Dolphin Imaging software v. 11.5 Premium.

After this standardization, reconstructions were also used to determine sagittal and axial sections. Reference lines were then identified, with the purpose of standardizing images so that the linear dimensions of the nasal cavity, before and after expansion, could be measured. Initially, in sagittal slices, the vertical reference line was placed over the anterior nasal spine, and the horizontal reference line was placed on the most inferior portion of nasal cavity (Fig 1). In axial sections, the reference line was placed along the median palatine suture, indicating its exact location. Afterwards, also in sagittal sections, the vertical reference line was placed over the posterior nasal spine, and the horizontal reference line was also placed on the most inferior portion of the nasal cavity (Fig 1). Thus, coronal sections could be generated and measurements taken both in the most anterior and in the most posterior regions of nasal cavity. 

**Figure 2:**
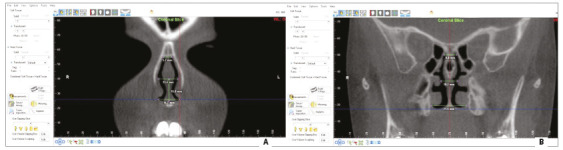
Determination of linear limits of the nasal cavity, in superior, middle and inferior portions, on coronal sections of (A) anterior and (B) posterior regions.

In coronal sections, specific tools of the software were selected to measure the desired distances. The area of interest was chosen by moving the reference lines, with the horizontal line being placed over the nasal cavity base, and another vertical line, which had previously been positioned, in axial sections, in the median sagittal region. The main marker was placed on the meeting point between these two lines. Then, the horizontal reference line was repositioned to the nasal cavity top, and a new marker was placed on the intersection of these lines, determining the height of the nasal cavity. The vertical reference line was then repositioned to determine the lateral limits in the superior, middle and inferior portions of the nasal cavity, and new markers were placed in these regions to measure the linear distances. After obtaining all measurements in the most anterior region, measurements in the most posterior region were taken (Fig 2). 

Values obtained in each measurement were compiled using the Microsoft Office Excel 2010 (Microsoft Office 2010, Washington, WA, USA), and analyzed using the SPSS version 16.0 (SPSS Inc., Chicago, IL, USA). Initially, a descriptive analysis was made (mean and standard deviation) with the purpose of identifying the general and specific characteristics of the studied sample. All the measurements were taken by the same evaluator, duly calibrated. Eight coronal sections (10% out of a total of 80 images), randomly selected, were examined once again after 15 days, and used to calculate the causal error for all the variables, by means of the Lin’s concordance coefficient, in order to verify the intra-examiner agreement (confidence interval of 95%), and systematic error. An index higher than 0.93 was obtained for all variables. Data distribution normality was verified with the Shapiro-Wilk test. Differences between T_0_ and T_1_ values were calculated for all the measurements using the Spearman correlation and paired Student’s *t*-test, with a significance level of 5%. 

## RESULTS

**Figure 3:**
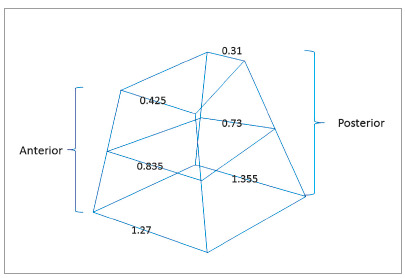
Representation of the opening pattern of anterior and posterior regions, and superior, middle and inferior portions, of the nasal cavity after RME.

The mean, standard deviation and *p*-value for measurements of each region are demonstrated in Table 2, in both T_0_ and T_1_ phases. It is possible to observe that all measurements showed a significant increase. In Table 3 and Figure 3, differences between T_1_ and T_0_, in the different measured regions, are shown. The Spearman correlation test revealed an index of 0.989, considered a strong correlation.

**Table 2:** Mean, standard deviation and p-value for each region, pre (T_0_) and post-RME (T_1_) phases.

Region	Times				p-valor
	T_0_		T_1_		
	Mean (mm)	Standard deviation (mm)	Mean (mm)	Standard deviation (mm)	
Anterior					
Superior	6.235	1.263	6.605	1.047	0.044*
Middle	12.675	1.452	13.54	1.796	<0.001*
Inferior	14.820	1.979	16.175	2.228	<0.001*
Posterior					
Superior	9.765	5.689	10.105	5.893	0.011*
Middle	17.500	5.961	18.230	6.056	0.008*
Inferior	18.575	5.477	20.005	5.559	<0.001*

## DISCUSSION

Studies conducted over the course of many years about possible alterations in nasal cavity resulting from RME were based on occlusal or lateral and frontal cephalometric radiographs.^1,5,7,8,11,13,15,17-19^ These studies state that the width variation of the nasal floor is about 0.4 mm to 6.5 mm. However, they should be considered with caution, because they were based on two-dimensional images with a great amount of superimpositions. Most recent research has been conducted using three-dimensional imaging,^10^ ^,^ ^12^ ^,^ ^24^ ^,^ ^25^ ^,^ ^27^ ^-^ ^33^ ^,^ ^36^ ^-^ ^38^ but few have evaluated changes in linear dimensions of the nasal cavity.^10^ ^,^ ^36^ In this research, the most inferior part of the nasal cavity showed an increase in width from 0.3 mm to 3.8 mm. These values are lower than that cited previously, but the greater precision and richness of details of tomographic images may explain these differences. In addition, although some studies have evaluated skeletal and volumetric changes after RME using CBCT,^10^ ^,^ ^12^ ^,^ ^21^ ^,^ ^23^ ^-^ ^27^ ^,^ ^29^ ^-^ ^31^ ^,^ ^34^ ^-^ ^38^ ^,^ ^40^ none have evaluated the changes in linear dimensions. 

Opening of the median palatine suture during RME, both in coronal and axial views, has been shown in various studies^2^ ^-^ ^4^ to be triangular in shape, with its vertex facing towards the nasal cavity in the coronal view, and towards the posterior nasal spine in the anteroposterior direction. This triangular configuration is controversial in the literature, because some studies have shown that the opening occurs with the suture edges almost parallel to each other.^16,31,39^ Recently, authors in two systematic reviews^33^ ^,^ ^40^ concluded that there is no consistent evidence for the pattern of opening of the median palatine suture, whether parallel or triangular. However, these findings refer to studies conducted considering the bony part of the palate of the oral cavity, and no studies have been found in which this evaluation was performed directly on the floor of the nasal cavity. In this research, in coronal views, it was possible to verify a great transverse movement in the inferior portion of the lateral walls of the nasal cavity, both in anterior and posterior sections, and consequent distancing of the nasal conchae in relation to the nasal septum. This improvement in linear dimensions was gradually decreased towards the frontonasal suture (Fig 3), with values of approximately 1.3 mm, 0.8 mm and 0.4 mm, in anterior sections, and 1.4 mm, 0.7 mm and 0.3 mm, in posterior ones (Table 3). This is in accordance with the literature, which states that in coronal view the opening occurs in a triangular form, with the apex facing towards the frontonasal suture.

**Table 3:** Linear changes (mm) between pre- and post-RME phases (T_1_-T_0_).

	T_1_-T_0_	
	Region	Difference (mm)
Anterior	Superior	0.425
	Middle	0.835
	Inferior	1.27
Posterior	Superior	0.31
	Middle	0.73
	Inferior	1.355

However, in axial sections, the opening pattern seems to be slightly different. The most inferior portion of the anterior and posterior regions presented a similar gain in the transverse direction, 1.3 mm and 1.4 mm, respectively (Table 3). These values indicate that the movement of the lateral walls of the nasal cavity was uniform (Fig 3), suggesting that the suture opening was parallel, as shown by other authors.^16^ ^,^ ^31^ ^,^ ^39^ Although these changes are statistically significant, they are not clinically relevant for orthodontics, particularly at the higher portions of the nasal cavity.

It is worth pointing out that, according to the literature,^7^ ^-^ ^11^ ^,^ ^17^ ^-^ ^19^ although the gain at the inner dimensions of the nasal cavity after RME procedure may be significant, its use for respiratory purposes only is not justified. Recently published studies^12^ ^,^ ^13^ showed an improvement in the respiratory pattern in mouth breathers. However, there is no scientific evidence indicating that children treated with expansion preserve the respiratory benefits acquired after a follow-up period, thus emphasizing the need for further long term studies.

## CONCLUSION

In view of the foregoing, it is possible to conclude that RME was effective in significantly increasing the internal dimensions of the nasal cavity, both in the anterior and posterior regions, and both in the inferior and superior portions, with two different patterns. In the coronal view, opening is triangular in shape, with its vertex facing towards the frontonasal suture. In the axial view, there is a uniform movement of the lateral walls, especially in the most inferior portion.
